# *In vivo* functions of miRNAs in mammalian spermatogenesis

**DOI:** 10.3389/fcell.2023.1154938

**Published:** 2023-05-05

**Authors:** Jian Chen, Chunsheng Han

**Affiliations:** ^1^ State Key Laboratory of Stem Cell and Reproductive Biology, Institute of Zoology, Chinese Academy of Sciences, Beijing, China; ^2^ Institute for Stem Cell and Regeneration, Chinese Academy of Sciences, Beijing, China; ^3^ Beijing Institute for Stem Cell and Regenerative Medicine, Beijing, China; ^4^ Savaid Medical School, University of Chinese Academy of Sciences, Beijing, China

**Keywords:** miRNA, spermatogenesis, mammal, *in vivo*, male fertility

## Abstract

MicroRNAs (miRNAs) are believed to play important roles in mammalian spermatogenesis mainly because spermatogenesis is more or less disrupted when genes encoding key enzymes for miRNA biogenesis are mutated. However, it is challenging to study the functions of individual miRNAs due to their family-wise high sequence similarities and the clustered genomic distributions of their genes, both of which expose difficulties in using genetic methods. Accumulating evidence shows that a number of miRNAs indeed play important roles in mammalian spermatogenesis and the underlying mechanisms start to be understood. In this mini review, we focus on highlighting the roles of miRNAs in mammalian spermatogenesis elucidated mainly by using *in vivo* genetic methods and on discussing the underlying mechanisms. We propose that studies on the roles of miRNAs in spermatogenesis should and can be conducted in a more fruitful way given the progress in traditional methods and the birth of new technologies.

## Introduction

**FIGURE 1:**
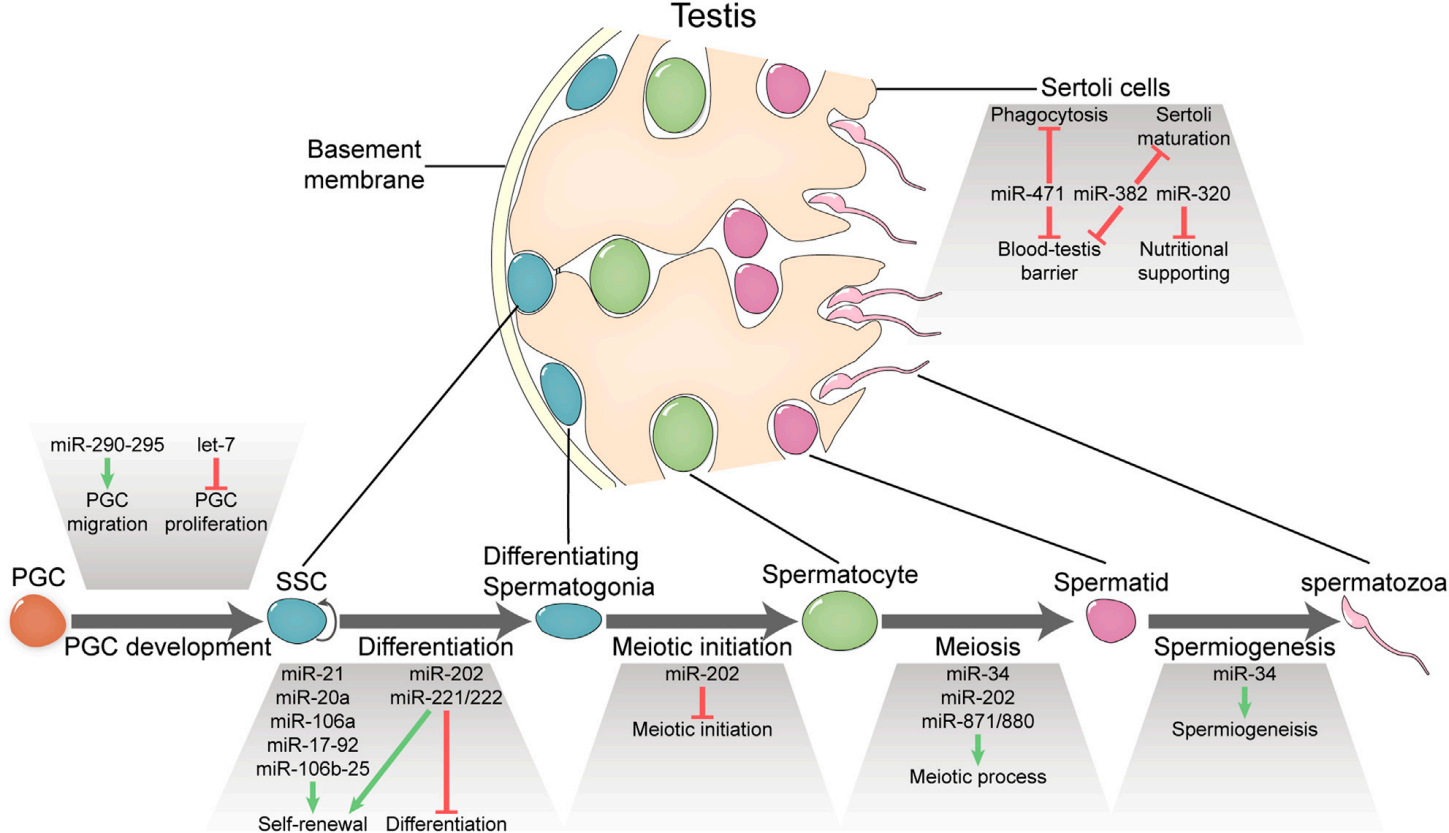
Functional miRNAs involved in spermatogenesis and male fertility. MicroRNAs portray essential or important roles in PGC development, spermatogenesis, and Sertoli functions.

Spermatogenesis is a cellular developmental process starting from the differentiation of spermatogonial stem cells (SSCs) and ending up with the production of spermatozoa via the sequential generation of many intermediate cell types including differentiating spermatogonia, meiotic spermatocytes, and haploid spermatids (Figure 1) (Handel and Schimenti, 2010; Griswold, 2016). Like other developmental processes, spermatogenesis is driven by the dynamic gene expression that is regulated at different levels along the path depicted by the central dogma. One of the unique features of spermatogenesis is that the complexity of transcriptome in spermatogenic cells is the highest compared with those in other cell types (Handel and Schimenti, 2010; Xia et al., 2020). Moreover, a large proportion of transcripts including mRNAs, miRNAs, piRNAs, lncRNAs, and circular RNAs are specifically expressed in spermatogenic cells (Soumillon et al., 2013; Lin et al., 2016). However, to what extent all these transcripts are functional remains an open question.

MicroRNAs are small RNAs that are evolutionarily conserved and play important roles in many developmental processes of metazoans (Bartel, 2018). Conventionally, primary miRNA transcripts are transcribed by RNA polymerase II and then processed by two RNase III enzymes, DROSHA and DICER, into mature miRNAs to evoke mRNA degradation and translational repression (Czech and Hannon, 2011). Global miRNA loss caused by mutations in key miRNA-biogenesis regulators generates severe defects in almost all tissues examined, whereas individual miRNA deletion often lacks dramatic phenotypic consequences, likely due to the functional redundancy of miRNAs (Park et al., 2012). miRNAs are believed to be also important for mammalian spermatogenesis as indicated by the disrupted spermatogenesis in mice that are mutants for genes encoding key enzymes involved in miRNA biogenesis (Hayashi et al., 2008; Maatouk et al., 2008; Korhonen et al., 2011; Romero et al., 2011; Greenlee et al., 2012; Wu et al., 2012; Zimmermann et al., 2014; Modzelewski et al., 2015; Hilz et al., 2016). Out of the 1,200 miRNAs that have been identified in mice, at least 300 are expressed in at least one of three spermatogenic cell types—type A spermatogonia, pachytene spermatocytes, and round spermatids (Chen et al., 2017).

It has been challenging to define the functions of these miRNAs in spermatogenesis for two reasons. First, most miRNAs are functionally redundant as predicted by high sequence similarities of miRNAs belonging to the same family. Second, miRNA genes usually distribute in the genome as clusters. Both features of miRNAs prevent effective investigation of their functions using traditional genetic perturbation method. Many studies about miRNA functions in spermatogenesis take indirect methods. Some infer miRNA functions from their expression patterns and/or their predicted targets while others are based on results in cultured cells. It is very suspicious how reliable conclusions based on such indirect methods can be extrapolated to *in vivo* conditions.

In this mini review, we summarize observations on miRNA functions at different stages of mammalian spermatogenesis and the underlying mechanisms if available derived from studies using gene knockout/overexpression mice and *in vitro* cultured SSCs upon being transplanted (Figure 1; Table 1). As spermatogenic cells are progeny of primordial germ cells (PGCs) (Saitou et al., 2012), we also include studies about miRNA function in PGCs. At the end, we discuss how miRNA functional studies can be conducted more effectively with traditional method and new techniques such as CRISPR-based procedures.

**TABLE 1:** Abnormal phenotypes observed in spermatogenesis in mice after knocking out or overexpressing one or more members of miRNA family/cluster.

Family/Cluster	miRNA(s) altered	*In vivo* study method	Function
miR-290 cluster	miR-290-295	Mouse conventional gene knockout	miR-290-295 promotes primordial germ cell migration Medeiros et al. (2011).
let-7 family	let-7g	Mouse embryonic overexpression (doxycycline induction)	let-7 reduces the primordial germ cell proliferation Shinoda et al. (2013).
let-7 family	miR-202	Mouse conventional gene knockout	1) miR-202 maintains stem cell pool Chen et al. (2017); 2) miR-202 prevents precocious spermatogonial differentiation and meiotic initiation Chen et al. (2021); 3) miR-202 safeguards meiotic progression Chen et al. (2022).
miR-21 family	miR-21	Mouse SSC transplantation (miRNA inhibitor treatment)	miR-21 maintains the self-renewal of mouse spermatogonial stem cells Niu et al. (2011).
miR-17 family	miR-20a and miR-106a	Mouse SSC transplantation (miRNA inhibitor or mimic treatment)	miR-20a and miR-106a promotes spermatogonial stem cell renewal (He et al., 2013).
miR-221/222 cluster	miR-221/222	Mouse SSC transplantation (miRNA inhibitor treatment or overexpression)	miR-221/222 maintains the undifferentiated state of spermatogonia (Yang et al., 2013).
miR-17-92 cluster (Mirc1)	miR-17-92	Mouse conditional gene knockout (*Vasa*-cre); tamoxifen-inducible gene knockout	miR-17-92 promotes normal spermatogenesis and spermatogonial stem cell self-renewal Tong et al. (2012) and Xie et al. (2016).
miR-106b-25 cluster (Mirc3)	miR-106b-25	Mouse conventional gene knockout	miR-106b-25 promotes normal spermatogenesis and early spermatogonial progression Hurtado et al. (2020).
miR-506 family	miR-871/880	Mouse conventional gene knockout	miR-871/880 safeguards the meiotic progression Ota et al. (2019).
miR-15/16 family	miR-15b	Transgenic mouse with spermatogenic cell-specific overexpression (*Pgk2* promoter)	Appropriate level of miR-15b safeguards spermatogenesis Chen et al. (2019).
miR-10 family	miR-10a	Transgenic mouse with spermatogenic cell-specific overexpression (*Ddx4* promoter)	Low level of miR-10a safeguards spermatogonial differentiation and meiotic process Gao et al. (2019).
miR-34 family	miR-34b/c/449	Mouse conventional double knockout for miR-34b/c and miR-449 loci (DKO)	1) miR-34 safeguards meiotic progression Comazzetto et al. (2014); 2) miR-34 is essential for later stages of spermiogenesis Comazzetto et al. (2014), Song et al. (2014) and Wu et al. (2014); 3) miR-34 is required for the formation of motile cilia in efferent ductules needed to transport immotile sperm from the rete testis to the epididymis Yuan et al. (2019).
miR-506 family	miR-471	Transgenic mouse with Sertoli cell-specific overexpression (*Rhox5* promoter)	Low level of miR-471-5p ensures efficient clearance of apoptotic germ cells in Sertoli cells, to maintain fertility Panneerdoss et al. (2017).
miR-154 family	miR-382	Transgenic mouse with Sertoli cell-specific overexpression (*Rhox5* promoter)	The decline in miR-382-3p in Sertoli cells of pubertal mice warrants blood-testis barrier formation and triggers the onset of spermatogenesis Gupta et al. (2021).
miR-320 family	miR-320	Mouse testis injection with miRNA mimic	miR-320-3p negatively regulates lactate production of Sertoli cells, ensuring the development of spermatocytes and spermatids Zhang et al. (2018).

## miRNAs involved in PGC development and SSC fate decision

The first piece of evidences implying miRNA functions in germ cells came from a study by Hayashi et al., in which the *Dicer1* was conditionally deleted using *Tnap*-Cre as the deletion initiator (Hayashi et al., 2008). In that study, poor proliferation of PGCs and spermatogonia was observed in the knockout mice. As the first direct evidence for the role of miRNAs in germ cell development, Medeiros et al. used conventional gene targeting method to delete the mammalian-specific miR-290-295 gene cluster, from which miRNAs are specifically expressed in early embryos and embryonic germ cells, and found that the number of post-migratory PGCs were reduced in both male and female mice mostly likely due to mislocalization of a subpopulation of migrating PGCs although the effect of a slower proliferation kinetics could not be excluded. Interestingly, female but not male fertility is compromised because male germ cells are able to recover from this initial germ cell loss due to their extended proliferative lifespan while female ones are unable to recover due to the lack of the mitotic amplification phase in fetal gonads (Medeiros et al., 2011).

let-7 family miRNAs are highly conserved in bilaterian animals and play important roles in many developmental and disease processes (Roush and Slack, 2008). The involvement of let-7 in germ cells development is first inferred by the roles of its negative regulator LIN28 in PGC proliferation (West et al., 2009), and then validated by the effect of induced overexpression of let-7 in transgenic mice (Shinoda et al., 2013). More specifically, LIN28A protein is detected in both gonocytes and undifferentiated spermatogonia and its conditional gene knockout in germ cells using *Ddx4*-Cre compromises PGC proliferation, the size of germ cell pool, and fertility in both male and female mice. Consistently, overexpression of let-7 in the doxycycline inducible iLet-7 mice also reduces the germ cell pool (Shinoda et al., 2013). These results suggest that the suppression of let-7 is important for PGC proliferation. These results are also consistent with earlier observations that let-7 directly regulates BLIMP1 (Nie et al., 2008), a key player in PGC specification and early development, and that BLIMP1 can rescue the effect of LIN28 deficiency during PGC development (West et al., 2009).

The intergenic miR-202 is distantly related to the let-7 family (Roush and Slack, 2008) and generates two complementary miRNAs, miR-202-5p and miR-202-3p, both of which are highly and specifically expressed in testes (Chen et al., 2017). By using an inducible CRISPR-Cas9 knockout platform in cultured SSCs and transplantation assays, we first showed that miR-202 is required to maintain stem cell pools. Using iTRAQ-based proteomic analysis and RNA sequencing, the target genes are identified and enriched with cell cycle regulators and RNA-binding proteins. Moreover, the RNA-binding protein RBFOX2, which is directly regulated by miR-202-3p, exhibits a regulatory role in meiotic initiation (Chen et al., 2017). Further, using knockout mice generated by the CRISPR-Cas9 technology, we showed that miR-202 prevents precocious spermatogonial differentiation and meiotic initiation by inhibiting the expression of key meiosis-initiating regulators—STRA8 and DMRT6. Moreover, *Dmrt6* mRNA is a direct target of miR-202-5p and *Dmrt6* knockdown could partially rescue the defects caused by miR-202 knockout in an *in vitro* assay. These findings add the miR-202/DMRT6 axis to the regulatory network of meiotic initiation (Chen et al., 2021). Notably, miR-202 knockout exerts no effects on Sertoli cell function (Chen et al., 2021).

Niu et al. first used high-throughput sequencing to compare miRNA profiles in Thy1^+^ SSC-enriched cells and Thy1^-^ somatic cell-enriched cells from pre-pubertal mice, and identified a panel of miRNAs that are preferentially expressed in the Thy1^+^ population. Out of this panel, miR-21 was selected for further study and its gene was found to be directly targeted and activated by transcription factor ETV5, which is critical for SSC self-renewal. More importantly, transient inhibition of miR-21 in cultured SSCs followed by transplantation assay indicated that miR-21 prevents germ cell apoptosis and maintains stem cell activity of the cultured SSCs (Niu et al., 2011). Using the same SSC perturbation/transplantation strategy, more miRNAs that maintain SSC/undifferentiated spermatogonium identity are reported (He et al., 2013; Yang et al., 2013). These include miR-20 and miR-106a belonging to miR-17 family originated from the miR-17-92 cluster that were initially found to be highly expressed in PGCs (Hayashi et al., 2008), and miR-221/222 from an X chromosome cluster. These studies also made effort to identify direct targets of miRNAs based on bioinformatics prediction and experimental validation. In this sense, *Stat3* and *Ccnd1* transcripts are likely direct targets of miR-20 and miR-106a (He et al., 2013) while *Kit* translation is directly suppressed by miR-221/222, indicating a role for miR-221/222 in preventing transition to a KIT^+^ differentiation state in spermatogonia (Yang et al., 2013).

The miR-17-92 cluster (Mirc1) and its paralogous clusters miR-106a-363 (Mirc2) and miR-106b-25 (Mirc3) generate miRNAs belonging to four families: the miR-17, miR-18, miR-19, and the miR-25 families (Mendell, 2008). These miRNAs, highly conserved among vertebrates, not only act as oncogenes to promote cell proliferation, suppress apoptosis of cancer cells, and induce tumor angiogenesis but also play essential roles during normal development of the heart, lungs, and immune system (Mendell, 2008). miRNAs from Mirc1 are highly expressed in PGCs and Thy1^+^ undifferentiated spermatogonia (Hayashi et al., 2008; Tong et al., 2012). Tong et al. deleted the Mirc1 cluster in mouse germ cells using *Vasa*-Cre and found that spermatogenesis is disrupted with reduced testis weight, lower sperm count, and appearance of Sertoli cell-only tubules (Tong et al., 2012). Xie et al. (2016) also showed that miR-17-92 knockout progressively results in smaller testes and reduced numbers of spermatogonia and SSCs, all of which are mediated by the activation of the mTOR-signaling pathway, indicating that Mirc1 miRNAs contribute to maintain the undifferentiated state of spermatogonia. A recent study demonstrates that the conventional knockout of the Mirc3 cluster results in similar phenotypes as the Mirc1 mutants in spermatogenesis, i.e., reduced testis size, altered spermatogenesis, and low sperm count (Hurtado et al., 2020). Interestingly, fertility of either Mirc1 or Mirc3 knockout mice is not significantly reduced compared with wild-type controls but is severely reduced in Mirc1^+/−^/Mirc3^−/−^ double knockout mice suggesting the dosage effect of miRNAs from these clusters (Hurtado et al., 2020).

## miRNAs involved in meiotic progression and postmeiotic development

Conditional knockout of *Dicer*, *Drosha* or *Dgcr8* in mouse germ lines after embryonic day 18 (driven by *Ddx4*, *Stra8* or *Ngn3* promoter) demonstrates that miRNAs are essential for meiotic progression, and spermiogenesis (Korhonen et al., 2011; Romero et al., 2011; Greenlee et al., 2012; Wu et al., 2012; Zimmermann et al., 2014; Modzelewski et al., 2015). Ota et al. identified three germ-cell specific miRNAs, miR-741-3p, miR-871-3p, and miR-880-3p, whose genes are contiguously located on the X chromosome, and generated single, double, and triple mutant mice (Ota et al., 2019). Spermatogenesis is normal in any of the three single mutant mouse lines but disrupted in the miR-871/880 double mutants and triple mutants. Numbers of undifferentiated spermatogonia and early-stage spermatocytes were not significantly changed, but incomplete meiosis arrest at the pachynema/diplonema transition was observed in the triple mutants. Surprisingly, a recent study reported that no phenotypes were observed when 18 out of the 21 X-linked miR-506 family miRNAs including the above three miRNAs were knocked out (Wang et al., 2020). This may be explained by the different mouse genetic backgrounds or counteracting effects of these miRNAs.

miR-202 is also involved in the regulation of meiotic progression. Loss of miR-202 in mice causes spermatocyte apoptosis and perturbation of the zygonema-to-pachynema transition. Multiple defects occur during meiosis prophase I in knockout mice, including disrupted synapsis and crossover formation, and inter-sister chromatid synapses. REC8 plays an essential role in homologous synapsis during meiotic prophase I and is cleaved by SEPARASE afterward. We provide evidence that miR-202-3p directly targets *Separase* mRNA, and miR-202 upregulates REC8 by repressing *Separase* expression (Chen et al., 2022). Therefore, miR-202 regulates meiotic progress by acting on the established SEPARASE-REC8 axis.

Several studies also used miRNA overexpression in transgenic mice to investigate their functions. For example, the overexpression of miR-15b, which is primarily expressed in spermatocytes, was reported to impair spermatogenesis (Chen et al., 2019). However, the affected cell types and stages were not clear. A similar study reported that overexpression of miR-10a in transgenic mice results in male sterility, meiotic arrest, and aberrant spermatogonial differentiation. The *Rad51* mRNA, which encodes a protein playing a critical role in meiotic recombination, was validated to be the direct target of miR-10a (Gao et al., 2019).

The members in the miR-34 family (miR-34a, 34b, 34c, 449a, 449b, and 449c) are conserved in vertebrates and are generated from three genomic loci—miR-34a, miR-34b/c, and miR-449, which are actively transcribed in the testes (Bouhallier et al., 2010; Bao et al., 2012; Concepcion et al., 2012). miR-34b/c and miR-449 are mainly expressed in pachytene spermatocytes and spermatids, whereas miR-34a is restricted to spermatogonia (Bouhallier et al., 2010; Bao et al., 2012; Concepcion et al., 2012). Surprisingly, mice with each of the three loci deleted and mice with both the miR-34a/miR-34b/c loci deleted revealed no discernible defects in spermatogenesis and fertility (Choi et al., 2011; Bao et al., 2012; Concepcion et al., 2012; Song et al., 2014). In contrast, oligoasthenoteratozoospermia and infertility were observed in mice with the miR-449/miR-34b/c double-knockout (DKO) or triple-knockout (Comazzetto et al., 2014; Song et al., 2014; Wu et al., 2014), indicating a redundant function of miR-449 and miR-34b/c in spermatogenesis. Interestingly, germ cell loss in DKO occurs at two distinct phases: during pachytene stage (Comazzetto et al., 2014) and later during spermatid elongation (Comazzetto et al., 2014; Song et al., 2014; Wu et al., 2014). Moreover, DKO leads to loss of the motile cilia in efferent ductules needed to transport immotile sperm from the rete testis to the epididymis (Yuan et al., 2019), reminiscent of previous observations that the formation of motile cilia in multiciliated epithelia is disrupted in DKO mice (Song et al., 2014; Wu et al., 2014; Yuan et al., 2015).

## miRNAs involved in Sertoli functions

Sertoli cells constitute a critical component in spermatogenesis and miRNA biogenesis is essential for Sertoli cells to mature, survive, and ultimately support spermatogenesis (Papaioannou et al., 2009; Kim et al., 2010). Several studies used transgenic mice to investigate the *in vivo* miRNA functions in Sertoli cells. miR-471 belongs to the miR-506 family (Wang et al., 2020), and miR-471-5p is highly expressed in Sertoli cells in an androgen-dependent manner. Overexpression of miR-471-5p in Sertoli cells increases germ cell apoptosis and impairs male fertility due to disrupted integrity of blood-testis barrier (BTB), and defective engulfment and clearance of apoptotic germ cells. miR-471-5p directly regulates Dock180 which interacts with autophagy member proteins to constitute a functional phagocytic complex, revealing an essential role for miRNAs in the efficient clearance of apoptotic germ cells by Sertoli cells (Panneerdoss et al., 2017). In rat, miR-382-3p level exhibits dramatic decline in pubertal Sertoli cells compared to infant ones. Prevention of the decline in miR-382-3p in Sertoli cells of pubertal mice leads to severe testicular defects, low epididymal sperm counts, and infertility due to defective BTB formation, impaired Sertoli maturation, and compromised androgen receptor signaling. This implies that the decline in miR-382-3p levels at puberty is essential for triggering the onset of robust spermatogenesis. Interestingly, miR-382-3p directly represses AR and WT1, which are crucial for functional competence of Sertoli cells (Gupta et al., 2021). miR-320-3p is exclusively expressed in mouse Sertoli cells, and miR-320-3p overexpression in Sertoli cells compromises male fertility and leads to oligozoospermia and reduced sperm mobility. These defects may be caused by dysregulation of the nutritional supporting function of Sertoli cells by reducing lactate production through directly inhibiting glucose transporter 3 (GLUT3) expression (Zhang et al., 2018).

## Discussion

Since the report of the first miRNA lin-4 in *C. elegans* in 1993, three decades have passed with big progresses in understanding the mechanism of biogenesis, the function, the underlying mechanism, and the regulated expression of miRNAs as a whole class of small RNAs (Bartel, 2018). These progresses are made by investigations using various cell types. Spermatogenic cells, possessing the most complex transcriptome and undergoing many unique processes, serve as ideal models to study gene regulation at different levels involving diverse regulators including miRNAs (Soumillon et al., 2013; Lin et al., 2016). Compared with other types of RNAs expressed in spermatogenic cells, miRNAs form a relatively smaller family and are supposed to be more tractable in their functional studies. However, as can be seen from the above review, we still know very little in every aspect.

We have only studied the function of a small number of miRNAs out of the more than 1,000 mammalian miRNAs using the mouse gene knockout/overexpression technique or the cultured SSC/transplantation method. The number of validated miRNA target genes is limited. And we know little about how miRNAs regulate biological processes through certain pathways—what pathways they regulate and through which pathways they are regulated. Despite the lack of in-depth knowledge about miRNAs, several vague patterns about their actions in spermatogenesis appear. The first one is that, like what has been observed in other biological processes, miRNAs in spermatogenesis also seem to be functionally redundant and their effects are minor and accumulative. Abolishment of the expression of a single miRNA seldom disrupts spermatogenesis, and if spermatogenesis is disrupted by perturbing the expression of several miRNA genes, the effect is still not dramatic and fertility is usually not affected. Second, miRNAs seem to execute functions more often during spermatogonial proliferation than during meiotic and post-meiotic development. This is consistent with what was observed that miRNAs are much more abundant in type A spermatogonia than in spermatocytes and spermatids in which piRNAs dominate (Gan et al., 2011). Third, miRNA targeted genes are functionally diversified compared with those of piRNAs, which are mainly transcripts of retrotransposons. Although piRNAs also target mRNAs for their regulated degradation, how general this theme remains to be further investigated (Gou et al., 2014; Zhang et al., 2015).

The challenges in studying miRNA function in spermatogenesis is not unique to spermatogenesis *per se*. In contrast, as can be seen from above review, spermatogenic cells possess several unique advantages for elucidating miRNA functions and the underlying mechanisms. First, SSCs can be amplified to a large population by long term culture, which provides sufficient materials for genetic manipulation and mechanistic studies. Second, cultured SSCs initiate meiosis by RA treatment or co-culture with Sertoli cells, providing a preliminary *in vitro* model for studying genes involved in meiotic initiation (Wang et al., 2016). Third, genetically modified SSCs can be transplanted into testes for *in vivo* phenotypic evaluation. This semi-*in vivo* method in miRNA studies not only is cost-effective but also allows researchers to focus on studying functions of genes in spermatogenesis without concerns brought about by traditional gene knockout techniques.

Notably, there are examples showing some contrasting findings regarding the phenotypic differences for miRNAs between knockdown and knockout models. For example, using knockout mice, Yuan et al. showed that miR-34b/c and miR-449a/b/c were not required for fertilization, first cleavage division, or subsequent development (Yuan et al., 2015), whereas Liu et al. revealed that injection of miR-34c inhibitor into zygotes significantly attenuated the first cleavage division after fertilization (Liu et al., 2012). The differences may come from the redundant manners of miRNA effects. The long-lasting fine-tuning functions of miRNAs could be compensated by other miRNA members in the knockout models, but not in the knockdown models, where miRNA inhibitors are usually designed to block the seed sequences of miRNAs and possibly inhibit all members of the families.

The early gene knockout method was mainly based on gene targeting technique. Perturbation of the expression of miRNAs, which are either closely clustered or transcribed as polycistronic units, is a truly challenging task. With the development of new technologies such as the CRISPR-based ones, this demanding work may be alleviated. For example, seed sequences of miRNA genes in a cluster can be separately targeted by carefully designed sgRNAs (Ota et al., 2019). As target identification and characterization of miRNA-mRNA networks are keys to elucidate the functions and mechanisms of miRNAs, the recent development of the high-resolution Halo-Enhanced AGO2 pull-down technique is promising in the new era of *in vivo* miRNA explorations (Li et al., 2020).
